# Surveillance of West Nile virus infections in humans and animals in Europe, monthly report – data submitted up to 24 June 2026

**DOI:** 10.2903/j.efsa.2026.10231

**Published:** 2026-07-15

**Authors:** European Centre for Disease Prevention and Control (ECDC), European Food Safety Authority (EFSA)

**Keywords:** West Nile virus, humans, birds, equids, outbreak.

## Abstract

Epidemiological summary

As of 24 June, two countries in Europe reported three locally acquired^1^ human cases of WNV infection: **Italy** reported two cases and **North Macedonia** one case. Dates of onset ranged from 12 to 27 May 2026. No deaths have been reported.

The cases were reported from three regions across the two countries. Although no cases had been reported by this time in 2025, some cases with onset dates in May and June were subsequently notified with a delay. Therefore, the current situation remains consistent with the early phase of the seasonal reporting pattern observed in previous years.

This year, human cases of WNV infection were reported for the first time ever in one affected area: **North Macedonia** in Vardarski (MK001).

Among the three cases reported this year, one person was aged 65 years or older, one was aged under 65 years, and the age of one person was unknown. All three cases were hospitalised. Neurological manifestations were reported in two individuals; information on clinical presentation was unavailable for the remaining case. As only three cases have been reported to date, comparisons with demographic or severity patterns observed over the previous decade are not yet meaningful. Further updates will be provided in subsequent monthly reports.

From the veterinary perspective, five WNV outbreaks have been reported in Europe in 2026: one among equids and four among birds. The equid outbreak was reported by **France** and started on 30 March 2026. The four bird outbreaks were reported by **Italy**, with start dates ranging from 31 March to 4 May 2026.

No information was available on the equid species involved in the outbreak reported in France in the Animal Disease Information System (ADIS). For birds, species information indicated that the four outbreaks reported in **Italy** involved hooded crows (three outbreaks) and a golden eagle (one outbreak).

Outbreaks in birds and/or equids have been reported in three regions across two countries. Both countries that reported outbreaks in 2026 had previously reported WNV outbreaks in birds and/or equids in the same regions, indicating that WNV is endemic in these areas.

The number of outbreaks in birds and equids reported during this first period of 2026 is similar to the mean monthly outbreak count for the same time of year, calculated for 2022–2025 for birds and for 2016–2025 for equids.

**Italy** reported both locally acquired human WNV cases and WNV outbreaks in birds; however, the human cases and bird outbreaks were reported from different regions.

Owing to delays in diagnosis and reporting, and because most WNV infections are asymptomatic or subclinical, the reported case numbers likely underestimate the true number of infections. Seasonal surveillance in humans primarily captures laboratory‐confirmed cases, which may further contribute to reporting delays.

Given the favourable weather conditions for WNV transmission in Europe, ECDC and EFSA expect further human cases and outbreaks in equids and birds to be reported in the coming weeks and months. In previous years, transmission has typically peaked in August and September.

ECDC and EFSA will continue to closely monitor the situation in Europe.

